# Forced migrant women’s Mental Health in the perinatal period in Germany- A mixed-method study

**DOI:** 10.1093/eurpub/ckac131.445

**Published:** 2022-10-25

**Authors:** R Al Munjid, L Teschemacher, E Mohr, M Engelhardt, J Breckenkamp, T Borde

**Affiliations:** 1 Alice Salamon Hochschule Berlin, Berlin, Germany; 2 Klinik für Gynakologie, Charité Universitatsmedizin, Berlin, Germany; 3 Epidemiologie und International Public Health, Universität Bielefeld, Bielefeld, Germany

## Abstract

**Background:**

After forced migration, pregnant women and new mothers face specific challenges in the host country. Little research focuses on the impact these challenges have on forced migrant (FM) women’s mental health (MH). The study’s aim shed light on FM women’s mental wellbeing in the perinatal period.

**Methods:**

For this mixed-method study 15 individual problem-centered interviews were carried out in Arabic with FM mothers living in Germany within the postnatal period to one year postpartum. The transcripts were analysed using the Framework Analysis. In addition, structured quantitative interviews with 3070 new mothers were conducted on 3 obstetric wards in Berlin over a period of 23 months using an adapted version of the MFMCQ and the PHQ4. FM women (n = 187) were compared with immigrant (n = 1192) and non-immigrant women (n = 1673). A Kruskal-Wallis-Test was performed to compare the three groups.

**Results:**

In the qualitative interviews mothers stated having depression and varied emotions (e.g. unavoidable sadness, relief). Most FM mothers indicated contextual factors (e.g. bad housing conditions) and structural barriers in perinatal healthcare as negatively impacting their MH. The preliminary quantitative analysis of the interviews conducted in birth clinics directly after birth showed no significant differences in the mean scores of the PHQ-4 within the compared groups (mFMW= 2.83; mimmi=2.61; mnonimmi= 2.5; p=.72).

**Conclusions:**

While the quantitative study part indicates that PHQ-4 scores are independent of FM experience, the qualitative part shows that FM new mothers face particular burdens in their living conditions that make them vulnerable to MH issues. Inconsistent results could be attributed to the different timing in which the structured questionnaires and qualitative interviews were conducted.

**Key messages:**

• To promote Mental health of new mothers, we advocate for a diversity oriented, responsive healthcare system.

• Effective approaches must be provided to include FM mothers in existing Early Childhood Intervention programs.

